# Sedentary Plant-Parasitic Nematodes Alter Auxin Homeostasis via Multiple Strategies

**DOI:** 10.3389/fpls.2021.668548

**Published:** 2021-05-28

**Authors:** Matthijs Oosterbeek, Jose L. Lozano-Torres, Jaap Bakker, Aska Goverse

**Affiliations:** Laboratory of Nematology, Department of Plant Sciences, Wageningen University and Research, Wageningen, Netherlands

**Keywords:** nematodes, auxin homeostasis, plant parasitism, IAA – indole-3-acetic acid, IBA – indole-3-butyric acid, indole propionic acid, PAA (phenylacetic acid), 4-Cl-IAA

## Abstract

Sedentary endoparasites such as cyst and root-knot nematodes infect many important food crops and are major agro-economical pests worldwide. These plant-parasitic nematodes exploit endogenous molecular and physiological pathways in the roots of their host to establish unique feeding structures. These structures function as highly active transfer cells and metabolic sinks and are essential for the parasites’ growth and reproduction. Plant hormones like indole-3-acetic acid (IAA) are a fundamental component in the formation of these feeding complexes. However, their underlying molecular and biochemical mechanisms are still elusive despite recent advances in the field. This review presents a comprehensive overview of known functions of various auxins in plant-parasitic nematode infection sites, based on a systematic analysis of current literature. We evaluate multiple aspects involved in auxin homeostasis in plants, including anabolism, catabolism, transport, and signalling. From these analyses, a picture emerges that plant-parasitic nematodes have evolved multiple strategies to manipulate auxin homeostasis to establish a successful parasitic relationship with their host. Additionally, there appears to be a potential role for auxins other than IAA in plant-parasitic nematode infections that might be of interest to be further elucidated.

## Introduction

The ability of many organisms to establish a long-term relationship with plants can result in the formation of unique specialised organs. For rhizobia, these are nodules on the roots of legumes that encapsulate the nitrogen-fixing bacteria (Oldroyd et al., 2011). For *Agrobacterium tumefaciens*, these are crown galls in plant stems that form a complex network to facilitate nutrient assimilation within the host (McCullen and Binns, 2006). The plant-parasitic cyst and root-knot nematodes follow a similar strategy to form a feeding structure inside the plant root to serve as a nutrient source (Abad and Williamson, 2010). These nematodes are obligate biotrophic endoparasites that infect a plant and subsequently manipulate the host machinery to facilitate pathogen survival. Plant-parasitic nematodes (PPNs) are a major agro-economic pest partly due to the lack of available effective countermeasures and their complex interaction with their host. Infections by PPNs result on average in a yield loss of 12.3% and cause yearly a financial harm of 157 billion dollars (Singh et al., 2015). Root-knot nematodes of the genus *Meloidogyne* and cyst nematodes of the genus *Heterodera* and *Globodera* are among the top 10 most harmful plant nematodes (Jones et al., 2013). The root-knot nematodes *Meloidogyne incognita* and *Meloidogyne javanica* are considered to be the most rapidly spreading among all pests and pathogens worldwide (Bebber et al., 2014).

Cyst nematodes (CN) emerge from their eggs as second stage juveniles (J2). They subsequently locate a suitable host and penetrate their root system. They move intracellularly through the root cortex and select an initial feeding cell near the vascular bundle by injecting a suite of secretory compounds. These compounds modify host cell metabolism, leading to a partial breakdown of plant cell walls and subsequent merging of neighbouring cells (Sobczak and Golinowski, 2011). This results in a syncytium that acts as a nutrient source for the nematode. Second stage juveniles of the root-knot nematodes (RKN) enter the root near the root tip and move intercellularly toward the apical meristematic region (Holbein et al., 2019). Near the apical meristem RKN turn around and migrate away from the root tip until they reach the differentiating vascular tissue. RKN select several cells in their vicinity, usually pro-vascular, to establish a feeding site upon injection of stylet secretions (Jones and Goto, 2011). Subsequently, these cells undergo several rounds of nuclear division and DNA replication that result in large multinuclear and hypertrophied cells. These are known as giant cells (GC) and serve as a nutrient source for the nematode. Typically, the RKN creates five to seven giant cells which can increase up to 100 times in size.

While the initiation and development of these feeding sites differ between RKN and CN, there are several striking similarities. Most notably, the accumulation of auxin at the site of infection at the onset of feeding site initiation. Auxin has long been suspected to play a crucial role during nematode infection (Sandstedt and Schuster, 1966). However, this had been difficult to study due to the absence of proper molecular methods. Due to the emergence of new techniques over the years, such as confocal microscopy and mass spectrometry more methods have become available to study the role of auxin during nematode infections. For example, reporter studies for both RKN and CN have shown the presence of auxin early on during feeding site formation (Absmanner et al., 2013). In addition, development of genetic tools and the establishment of Arabidopsis as a model plant for CN and RKN interactions has contributed to the study of plant hormones in nematode feeding sites (Sijmons et al., 1991). For example, it was shown that in the auxin-insensitive Arabidopsis mutant *axr2* CN infection is compromised (Goverse et al., 2000). Nevertheless, despite technological and biological advances no clear picture of the role of auxin in nematode infection is available.

Auxin is arguably the most important signalling molecule in plants and plays a key role in nearly all growth and developmental processes (Weijers and Wagner, 2016). In turn, a lot is known about how auxin synthesis, transport, and perception work in addition to the genes that are transcribed through auxin signalling (Zhao, 2010; Leyser, 2018). Auxin is often synonymously used with indole-3-acetic acid (IAA). However, auxin is actually a class of several hormones with similar functions. Several of these auxins can also activate classical reporter genes such as DR5-GUS (Sugawara et al., 2015). IAA is indeed the major auxin type known and a major player in many developmental processes such as cell division, root development and organogenesis. Despite the insight we have in these auxin-mediated processes our knowledge on its role during nematode infection remains incomplete.

In this review, we bring together the scattered information on the many aspects of auxin metabolism in feeding site initiation and development, from biosynthesis, catabolism and transport to perception and signalling. In addition, we discuss how PPNs may change auxin homeostasis. We first describe the current knowledge on auxin-related processes in plants and afterward explore how PPNs affect these processes upon infection. This is not only done for IAA but also for all other naturally occurring plant auxins. Here, we aim to provide an overview of the molecular and physiological mechanisms underlying the role of auxin homeostasis in feeding site formation in plant roots and to highlight new avenues to explore in future research.

## Auxins at Nematode Feeding Sites

In plants, five types of endogenous auxins are currently known: indole-3-acetic acid (IAA), 4-chloroindole-3-acetic acid (4-Cl-IAA), phenylacetic acid (PAA), indole-3-butyric acid (IBA), and indole-3-propionic acid (IPA). IAA has been widely recognised as the most influential auxin in regulating plant growth and development, but other endogenous auxins have been acknowledged to play various important roles. The auxin 4-Cl-IAA is synthesised by several plant species of the Fabeae and Trifoleae of the Fabaceae family and appears to affect fruit and seed development (Ernstsen and Sandberg, 1986; Ozga et al., 2009; Lam et al., 2015; McAdam et al., 2017). PAA is widespread among plants, occurs at higher concentrations than IAA, and is capable of regulating the same set of auxin-responsive genes through the TRANSPORT INHIBITOR RESPONSE1/AUXIN SIGNALING F-BOX (TIR1/AFB) pathway (Wightman and Lighty, 1982; Sugawara et al., 2015). IBA was originally thought to be a synthetic auxin until it was detected as an endogenously produced auxin. Although the underlying mechanism remains unknown, IBA strongly induces adventitious root formation at a higher rate than IAA (Epstein and Ludwig-Müller, 1993; Ludwig-Müller, 2000). IPA is speculated to be present in soybean and has also been found in *Cucurbita pepo* and *Pisum sativum* (Segal and Wightman, 1982; Schneider et al., 1985; Jiang et al., 2020). Additionally, IPA has been detected in Rhizobium root nodules of pea where it binds to horseradish peroxidase C (Badenoch-Jones et al., 1984; Veitch and Williams, 1990). While IAA undoubtedly will stay a focal point of research, the potential roles and functions of other endogenous plant auxins have gained increasing interest among developmental biologists (Ludwig-Müller, 2011; Simon and Petrášek, 2011; Frick and Strader, 2018; Cook, 2019).

**FIGURE 1:**
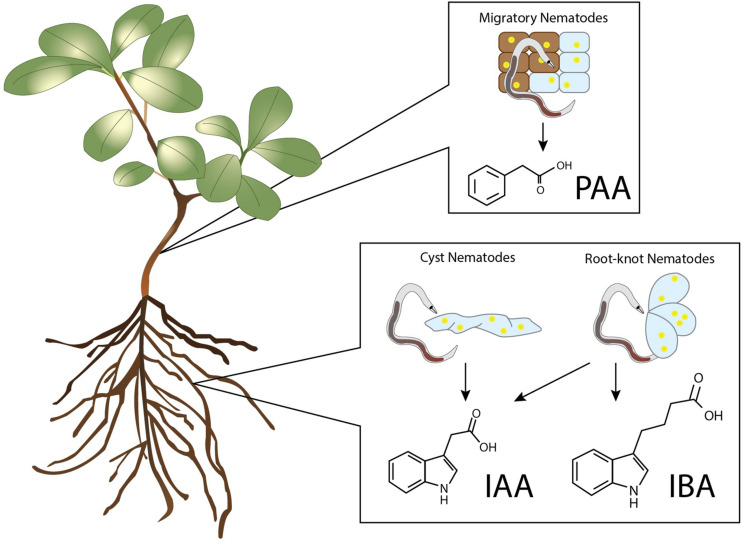
Auxins detected in nematode infected plant tissues. PAA was detected in callus upon infection by the necrotrophic plant-parasitic nematode *Bursaphelenchus xylophilus* (Kawazu et al., 1996; Zhang et al., 1997). Increased levels of IAA have been found in the infection sites of the cyst nematodes *Heterodera schachtii*, *H. cruciferae*, *H. trifolii*, and *Globodera rostochiensis*, and in the galls of the root-knot nematodes *Meloidogyne incognita*, *M. hapla*, and *M. javanica* (Yu and Viglierchio, 1964; Viglierchio and Yu, 1968; Hanschke et al., 1975; Agarwal et al., 1985; Ganguly and Dasgupta, 1987). Elevated levels of IBA were only found in the infection sites of the root-knot nematode *M. incognita* (Yu and Viglierchio, 1964).

Elevated levels of IAA and other auxins have been detected in nematode infection sites of various plant species and in some cases even in egg masses of nematodes (Yu and Viglierchio, 1964; Viglierchio and Yu, 1968; Hanschke et al., 1975; Agarwal et al., 1985; Ganguly and Dasgupta, 1987). These auxins were detected during infections by the RKN *Meloidogyne incognita*, *M. hapla*, *M. javanica*, and the CN *Heterodera schachtii*, *H. cruciferae*, *H. trifolii*, and *Globodera rostochiensis* (Figure 1). These auxins were measured using chromatography combined with the Avena first internode test, as well as Ehrlich and Salkowski chemical tests (Yu and Viglierchio, 1964). Thus, already in the early 60s a prominent role for auxins in nematode feeding site development was recognised. Besides IAA, IBA was exclusively detected in the galls of *M. incognita* (Viglierchio and Yu, 1968). IBA is thought to mainly act through conversion to IAA and contributes to root hair expansion, secondary root formation, and the auxin pool in the root apical meristem (Frick and Strader, 2018). As such, there is a potential role for IBA in feeding cell development, because lateral root formation and feeding cell development share similar developmental processes (Cabrera et al., 2015a). Infection of *M. incognita* as well as other RKNs and CNs lead to spontaneous secondary root formation originating from the feeding site to which auxins seems to contribute (Olmo et al., 2017; Levin et al., 2020). Whereas a role of auxin in CN and RKN parasitism has been widely accepted, this is not the case for PPNs with a migratory lifestyle. Therefore, it is interesting to note that PAA has been detected in callus tissue infected with *Bursaphelenchus xylophilus* (Kawazu et al., 1996; Zhang et al., 1997). This nematode inflicts the pine wilt disease and it has been suggested that PAA induces benzoic acid accumulation which results in the wilting of plant tissue (Kawazu et al., 1996). However, not all auxin types have been studied during PPN infection. To the best of our knowledge, no records have been published yet describing measurements of the auxins 4-Cl-IAA and IPA in infection sites of PPNs.

## Manipulation of *De novo* Auxin Biosynthesis in Nematode Feeding Sites

Elevated levels of auxins during nematode infection can be the result of various mechanisms, one of which is increased auxin biosynthesis. Indole moiety containing auxins (IAA, 4-Cl-IAA, IBA, IPA) are derived from tryptophan (Trp) while non-indole auxins such as PAA are presumably synthesised from phenylalanine (Cook et al., 2016). Biosynthesis of tryptophan and phenylalanine require the shikimate metabolic pathway, which is not present in animals, making these compounds essential amino acids. *De novo* IAA is synthesised from amino acids in the aerial part of the plant as well as specific regions in the root, such as the meristematic zone (Ljung et al., 2005). Several biosynthesis routes have been postulated for the synthesis of indole-3-acetic-acid. For Trp-dependent IAA synthesis four pathways are proposed: (1) the indole-3-acetamide (IAM) pathway, (2) the indole-3-pyruvic acid (IPyA) pathway, (3) the tryptamine (TAM) pathway, and (4) the indole-3-acetaldoxime (IAOx) pathway (Mano and Nemoto, 2012). Here, we discuss which auxin biosynthesis routes may be relevant for successful nematode infections.

**FIGURE 2:**
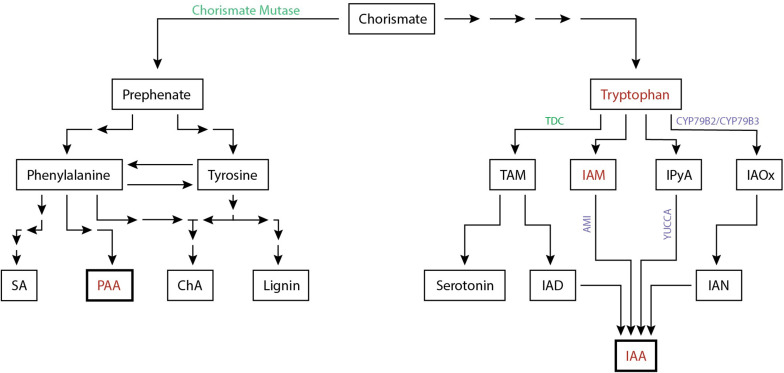
Simplified biosynthesis pathway of the auxins IAA and PAA in plants from the common precursor chorismate. Auxins and auxin precursors for which upregulation is shown are indicated (red). Enzymes involved in the biosynthesis pathway shown to be differentially regulated either at the protein level (green) or at the transcriptome level (purple) are depicted (Supplementary Table 1). The number of arrows indicate the number of conversion steps with four indicating four or more intermediate steps. Converging arrows show a shared intermediary compound. SA, salicylic acid; PAA, phenylacetic acid; ChA, chlorogenic acid; TAM, tryptamine; IAM, indole-3-acetamide; IPyA, indole-3-pyruvic acid; IAOx, indole-3-acetaldoxime; IAD, indole-3-acetaldehyde; IAN, indole-3-acetonitrile; IAA, indole-3-acetic acid.

All known auxin biosynthesis routes of IAA start with tryptophan although it is speculated that a non-Trp dependant pathway exists. Tryptophan concentrations increased by 53% in the feeding sites of *M. incognita* (Setty and Wheeler, 1968). In addition, the intermediate compounds indole-3-acetonitrile (IAN) can be found in galls of this nematode as well (Yu and Viglierchio, 1964). IAN is an intermediate compound of the IAOx biosynthesis pathway. The presence of this compounds suggests that local auxin biosynthesis occurs in feeding sites (Figure 2). This is supported by transcriptome analyses of CN and RKN infections showing that IAA biosynthesis genes are differentially expressed, such as *AMI*, *CYP79B2*, *CYP79B3*, and *YUCCA* (Supplementary Table 1). Of these differentially expressed auxin biosynthesis genes 76% (19/25) are upregulated upon *H. schachtii* or *M. incognita* infection of *A. thaliana*. Additionally, upregulation of YUC1, YUC3, YUC8, YUC10, and AMI1 support a role for the IAM and the IPyA pathway in local auxin biosynthesis. Although no intermediary compounds for these pathways are reported, modern day techniques may shed light on this. The transcriptome data together with the increased amount of tryptophan and IAN indicate that local auxin biosynthesis might occur during infection of plant roots by *H. schachtii* and *M. incognita*.

Aside from the IAOx, IAN, and IPyA pathway, the TAM biosynthesis route is affected as well during parasitism. The first step in the TAM biosynthesis pathway is the conversion of Trp to TAM and is catalysed by the enzyme tryptophan decarboxylase (TDC). TDC has been shown to be transcriptionally upregulated in feeding sites of the cyst nematode *H. avenae* and showed increased enzyme activity in that of *G. rostochiensis* (Giebel et al., 1979; Huang et al., 2018). TDC loss-of-function or overexpression mutants in *Aegilops variabilis*, a close relative to wheat, do not seem to affect IAA content during *H. avenae* infection despite its role in auxin biosynthesis (Huang et al., 2018). This is in accordance with experimental evidence showing a low contribution of the TAM pathway to the overall IAA pool in plants (Facchini et al., 2000). Furthermore, TDC regulates secondary metabolites rather than auxin biosynthesis suggesting that TDC has a secondary role in plant defence (Huang et al., 2018).

As discussed, changes in local auxin biosynthesis seem to occur during PPN infection. The nematode effector chorismate mutase (CM) is suspected to contribute to these changes. Although the PPN inject a suite of effectors into host plants, little is known about effectors directly targetting auxin biosynthesis, with the exception of CM. CM was first discovered in *M. javanica*, was later found to be widely distributed in both CN and RKN, and is currently used as a diagnostic marker for nematode parasitism (Lambert et al., 1999; Bekal et al., 2003; Doyle and Lambert, 2003; Jones et al., 2003; Huang et al., 2005; Long et al., 2006; Vanholme et al., 2009; Yu et al., 2011; Chronis et al., 2014). Upon discovery, it was suggested that CM alters plant cell development by negatively affecting auxin biosynthesis (Doyle and Lambert, 2003). Endogenous CM converts chorismate to prephenate in the shikimate pathway which occurs in plastids (Tohge et al., 2013). Coincidently, chorismate is also the branching point in the shikimate pathway from the biosynthesis of indole to the biosynthesis of phenylalanine and tyrosine. As such, the release of the effector chorismate mutase could lead to resource competition over chorismate and as a result, shift more to the synthesis of phenylalanine and tyrosine than to indole and by extension IAA (Doyle and Lambert, 2003). As phenylalanine and tyrosine are also amino acids, it is possible that the nematode uses chorismate mutase to increase nutrient production. Alternatively, the increase in phenylalanine via the shikimate pathway may enhance the production of the non-indole auxin PAA although its presence in feeding sites of CN and RKN remains to be elucidated. In addition, it is worthwhile noting that the plant defence hormone salicylic acid (SA) is also produced from phenylalanine. However, various data suggest that the secreted CM actually reduces the synthesis of the plant defence hormone salicylic acid (SA) (Djamei et al., 2011; Wang et al., 2018). Biosynthesis of SA occurs in plastids and uses the plastid fraction of chorismate, while a secreted CM would act on the cytoplasmic fraction of the chorismate pool. The secreted CM likely reduces the cytoplasmic fraction of chorismate leading to an increased flow from the plastids to the cytosol resulting in a depletion of the chorismate in the plastid fraction and therefore a decrease in SA biosynthesis.

Together, it can be concluded that nematodes seem to locally increase auxin biosynthesis through increasing precursor metabolites and increasing biosynthesis gene expression, thereby stimulating production of IAA most likely through the IAOx pathway. In addition, transcriptional analysis revealed that nematodes affect the IAN and IPyA pathway as well as the TAM pathway. Lastly, cyst and root-knot nematodes inject effectors e.g., chorismate mutase to reduce SA biosynthesis, which may have hypothetically an inhibitory effect on local IAA synthesis and a positive effect on PAA production (Figure 2).

## Manipulation of Auxin Catabolism in Nematode Feeding Sites

**FIGURE 3:**
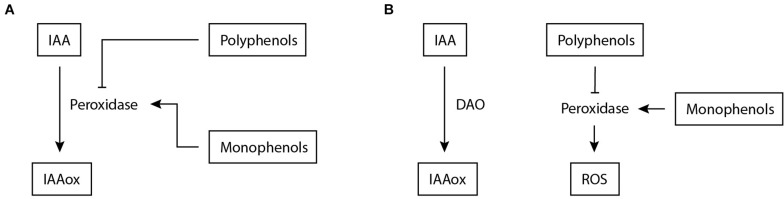
Proposed mechanisms involved in IAA inactivation during nematode infection of plants. **(A)** Previously, a mechanism was suggested for IAA inactivation during nematode infection based on (Giebel, 1970; Giebel, 1974; Giebel and Lamberti, 1977). Peroxidase is assumed to facilitate the conversion of IAA to IAAox, which is stimulated by increased monophenols levels and inhibited by polyphenols. **(B)** Based on new insights (Normanly et al., 1995), an alternative model for IAA inactivation is proposed. DAO facilitates IAA oxidation, but it is anticipated that additional inactivation mechanisms are involved. In this model, peroxidases remain regulated by phenols but now enhance ROS production as reported by Simonetti et al. (2010); Jin et al. (2011), and Sung et al. (2019). IAA, indole-3-acetic acid; IAAox, oxidized indole-3-acetic acid; DAO, DIOXYGENASE FOR AUXIN OXIDATION; ROS, reactive oxygen species.

An alternative strategy for nematodes to elevate auxin levels is to interfere with auxin breakdown. The main inactivation pathway known for IAA is oxidation to 2-oxindole-3-acetic acid (oxIAA) (Pěnčík et al., 2013). IAA oxidation has long been considered to be done through peroxidases. In addition, it was believed that IAA levels were affected by phenolic compounds via inhibition of peroxidase activity (Sági and Garay, 1961). This has been the prevailing view in plant nematology for many years as well. Various studies demonstrated that peroxidase activity is downregulated upon infection with CN and RKN (Giebel, 1970; Szczygiel and Giebel, 1970; Wilski and Giebel, 1971; Janas, 1976; Ganguly and Dasgupta, 1987; Molinari, 1991; Leela et al., 1993). A commonly used method to assess IAA oxidase activity upon nematode infection was to add horseradish peroxidases to root homogenates and measure O_2_ production (Giebel, 1970; Giebel, 1974; Giebel and Lamberti, 1977). These measurements revealed that phenolic compounds are key in regulating oxidase activity upon nematode infection (Figure 3A). Later on, it was shown that peroxidases do not play a significant physiological role as IAA oxidases in plants (Normanly et al., 1995; Ljung et al., 2002; Stepanova and Alonso, 2016).

Considering that peroxidases have no significant IAA oxidase activity *in planta*, the relation between phenols and nematode infection needs to be re-examined and thereto we propose a possible model in which phenol and peroxidase activity are uncoupled from auxin oxidation during infection (Figure 3). This model emerges from earlier studies that have shown that phenol content is often correlated with susceptibility to PPNs (Szczygiel and Giebel, 1970; Knypl and Chylinska, 1975; Giebel and Dopierala, 1982), where the polyphenolic fraction has an inhibiting effect on IAA oxidation and the monophenolic fraction a stimulating one. It was shown for *M. incognita* and *G. rostochiensis* that the ratio between mono- and poly-phenols correlates to susceptibility (Giebel, 1970; Yuan et al., 1996). A plausible function of peroxidases in mediating resistance (Simonetti et al., 2010; Jin et al., 2011; Sung et al., 2019) is the generation of reactive oxygen species (ROS). ROS stimulate cell wall reinforcement through lignification and extensin cross-linking and induce additional defence mechanisms (Almagro et al., 2009; Marhavý et al., 2019). Thus, phenol content correlates with PPN susceptibility and likely regulates peroxidase activity during infection. In this model, peroxidases do remain related to nematode infection, albeit not as IAA oxidases but as regulators of ROS (Figure 3B).

The discovery that peroxidases do not play a significant physiological role as IAA oxidases in plants led to the search for alternative candidates. In 2013, the characterisation of a male-sterile mutant in rice resulted in the identification of the candidate enzyme DIOXYGENASE FOR AUXIN OXIDATION (DAO) as a player in this oxidation mechanism (Zhao et al., 2013). Orthologs of this gene have subsequently been identified in Arabidopsis demonstrating the conserved nature of this gene between monocots and dicots (Mellor et al., 2016; Porco et al., 2016). Mutant and expression studies of DAO showed little impact on the general oxIAA pool, making it unlikely that DAO is the main component in this process (Stepanova and Alonso, 2016). Transcriptomic data shows that *AtDAO1* and *AtDAO2* in *A. thaliana* are upregulated upon CN and RKN infection (Supplementary Table 1). As DAO is likely not the only component in IAA oxidation we hypothesise that feeding sites may contain several redundant oxidation pathways that have yet to be identified.

## Conjugation of Auxins in Nematode Feeding Sites

An alternative mode of action for DAO to inactivate auxins in plants is through conjugation with an amino acid, sugar, or protein. The conjugated forms are presumed to function as either storage or intermediates destined for degradation (Woodward and Bartel, 2005). The conversion of an auxin to an ester conjugate with sugar involves UDP-glucose transferases, whereas conversion to an amide conjugate with amino acids requires IAA-amino acid conjugate synthetases (Ludwig-Müller, 2011). Conversion to an amide protein conjugate occurs through a still unknown mechanism. Beside IAA, all other auxins can be conjugated in this manner (Bajguz and Piotrowska, 2009). Although auxins can be conjugated to nearly all amino acids, their functions differ. IAA conjugates IAA–Asp and IAA–Glu are considered precursors for a degradation pathway while IAA-Trp acts as an inhibitor of auxin biosynthesis (Staswick, 2009). Additionally, a small fraction of auxin amino acid conjugates act as storage forms and can be hydrolysed back to their unbound form via auxin amino acid conjugate hydrolases (LeClere et al., 2002).

These processes seem to be affected during nematode infection at various time points. The GRETCHEN HAGEN 3 (GH3) family is such an example that is active in white clover (*Trifolium repens* cv. Haifa) during the initiation of root galls by *M. javanica* (Hutangura et al., 1999). The GH3 family is co-responsible for regulating the activity of IAA, SA, and JA and consists of three phylogenetic groups. The second group consists of IAA-amino acid conjugate synthetases and is one of the primary auxin-response genes (Zhang et al., 2018). As such, these GH3 genes are responsible for creating IAA-amino acid conjugates for either storage or degradation purposes. Transcriptomic data suggest that several GH3 genes are expressed in the feeding sites of cyst and root-knot nematodes (Beneventi et al., 2013; Supplementary Table 1). GH3.1, GH3.3, GH3.4, GH3.15, GH3.17 are upregulated during *H. schachtii* and/or *M. incognita* infection of *A. thaliana*, suggesting that GH3 genes act as a positive regulator in feeding site development (Supplementary Table 1). While upregulated at early stages of infection, a reporter study in white clover (*Trifolium repens* cv. Haifa) with *M. javanica* shows that GH3:gusA expression disappears after 72 h post-inoculation (Hutangura et al., 1999). This suggests that GH3 genes may play a role in regulating hormone levels during early feeding site initiation and afterward might be downregulated to maintain high levels of free active IAA. The biological significance of GH3 in RKN infection is supported by the overexpression mutant of *GH3.1* in *Oryza sativa*, which decreases the infection by *M. graminicola* (Yimer et al., 2018).

So, conjugation of auxins may contribute to fine tuning IAA levels during the development of feeding sites induced by sedentary nematodes like *M. javanica* and *M. graminicola*. The temporal variation in GH3 expression levels seems to be required for successful infections. However, current data on the differential expression of conjugating enzymes is limited to a few root-knot nematodes and it remains unknown how representative this dataset is, whether IAA conjugates are indeed created in the feeding site, and whether other auxins see a similar fate.

## Auxin Transport in Nematode Feeding Sites

Next to auxin anabolism and catabolism, transport processes are key in the accumulation of auxins at specific locations. IAA is transported as either a general unregulated bulk flow of auxin through mature phloem or through an actively regulated carrier-mediated cell-to-cell directional transport called active polar auxin transport (PAT) (van Berkel et al., 2013). PAT is mediated by a set of specific efflux and influx carriers. In Arabidopsis, the transmembrane proteins of the AUX1/LIKE AUX1 (AUX1/LAX) family act as influx carriers. Efflux occurs through the PIN-FORMED (PIN) proteins and the ATP-binding cassette subfamily B (ABCB)-type transporters of the multidrug resistance/phosphoglycoprotein (ABCB/MDR/PGP) protein family (Cho and Cho, 2013; Adamowski and Friml, 2015). Of the PIN protein family PIN5, PIN6, and PIN8 are presumed to regulate IAA transport between the endoplasmic reticulum and the cytosol and are suspected to regulate free IAA levels in the cytoplasm. The other PIN proteins (PIN1, PIN2, PIN3, PIN4, PIN7) are localised at the plasma membrane and facilitate cellular auxin efflux (Krecek et al., 2009). Five members of the ABCB subfamily have so far been associated with auxin transport: ABCB1, ABCB4, ABCB14, ABCB15, and ABCB19. The ABCB proteins localise to the plasma membrane of the cell in a non-polar manner and maintain auxin homeostasis providing a stable and uniform auxin distribution among cells (Cho and Cho, 2013).

Transport proteins for the auxins IPA and PAA have yet to be discovered. However, PAA forms concentration gradients in maize coleoptile similar to IAA (Sugawara et al., 2015). These PAA gradients are unaffected by the PIN transport inhibitor NPA and do not change when subjected to gravitropism stimuli. From this it was concluded that PAA is not transported actively and directionally in plants. As such PAA has distinct transport characteristics compared to IAA and it was speculated that the formation of the PAA gradient might be attributed to regulation of local biosynthesis. The exact transport proteins of 4-Cl-IAA are unknown, but an auxin transport competition assay suggests that IAA influx and efflux proteins transport 4-Cl-IAA in addition to IAA (Simon et al., 2013). In the case of IBA some transporters have been identified. Its efflux is promoted by ATP-BINDING CASSETTE G36/PLEIOTROPIC DRUG RESISTANCE 8/PENETRATION 3 (ABCG36/PDR8/PEN3) and ABCG37/PDR9/PIS1 (Strader and Bartel, 2009; Růžička et al., 2010). Although IBA uptake is a saturable process it is suspected to be transported across long distances in the plant mainly in a conjugated form (Rashotte et al., 2003).

Polar auxin transport is important for induction and development of nematode feeding sites and contributes to the local accumulation of auxin upon infection. This was already shown in 1972, where removal of the shoot tip from *Lycopersicon esculentum* resulted in a sharp decrease in infections of *G. rostochiensis* (Steinbach, 1972). Additionally, the disruption of PAT through inhibition with N-(1-naphthyl)phthalamic acid (NPA) during *G. rostochiensis* infection resulted in abnormal syncytium development (Goverse et al., 2000). PAT is significantly altered during feeding site development by both cyst and root-knot nematodes. Reporter studies have shown that during infection of *H. schachtii* PIN1 and PIN7 are absent from the feeding site suggesting a strong decrease in downward auxin flow (Grunewald et al., 2009). Interestingly, PIN3 and PIN4 seem to be highly expressed and specifically localise toward the lateral sites of the feeding site. During RKN infection PIN3 seems to be active during early infection in neighbouring cells at the basipetal side of the gall but not inside the feeding site. However, PIN3 expression changes at later stages where it also becomes active in giant cells. PIN1 seems to be highly expressed at the basal site of young galls whereas PIN7 expression seems to be suppressed. This suggests that in giant cells the downward auxin flow is stemmed and laterally redistributed.

PIN localisation is tightly controlled and influenced by multiple factors. An intriguing example of this is the cyclophilin A ortholog *DIAGEOTROPICA* (*DGT*) gene in *Solanum lycopersicum* (Goverse et al., 2000). The *DGT* mutant abolishes the organogenesis of lateral roots and affects PIN expression and localisation to the plasma membrane at the root tip (Ivanchenko et al., 2015). This has been shown in an expression study where *PIN2* transcripts decrease in the *dgt* mutant and the localisation of PIN1 and PIN2 is affected when *DGT* is expressed. When infecting a tomato *dgt* mutant with *G. rostochiensis*, inhibition of nematode development was observed, suggesting that the regulation of *PIN1* and *PIN2* through *DGT* is necessary for the development of nematode feeding sites (Goverse et al., 2000). The auxin efflux protein PIN2 is not only regulated by DGT, but additionally seems to be the target of several hormonal signalling pathways that control its localisation during nematode infection. PIN2 is also known as ETHYLENE INSENSITIVE ROOT 1 (EIR1) and its gene was first identified as a knockout plant insensitive to ethylene which later was recognised as PIN2. A PIN2 mutant in *A. thaliana* showed decreased susceptibility to *H. schachtii* infection (Goverse et al., 2000; Wubben et al., 2001). Additionally, PIN2 localisation is affected by a novel hormone class known as strigolactones (SL) that function as branching inhibitors. It has been shown that the SL deficient mutant *max4-1* and the SL signalling mutant *max2-1* have enhanced nematode infection due to the formation of enlarged feeding cells possibly through interaction with PIN2 (Escudero Martinez et al., 2019). In addition to ethylene and strigolactones, the cytokinin hormones could affect PIN localisation during nematode infection as well. Cytokinins accumulate in feeding sites and are essential for proper nematode development (Lohar et al., 2004; Siddique et al., 2015). Interestingly, it has been shown that *H. schachtii* can produce cytokinins that affect infection (Siddique et al., 2015). Cytokinins are known to affect the localisation of PIN1, PIN3, and PIN7 but whether this occurs during nematode infection remains unknown (Bishopp et al., 2011; Marhavý et al., 2014; Šimášková et al., 2015; Waldie and Leyser, 2018). Interestingly, PIN3, but not other PIN proteins, can also be induced by the hormone PAA, suggesting that PAA might also be a regulator of PIN expression (Sugawara et al., 2015).

The CN infection process goes along as well with an increased expression of the AUX1 importer in young feeding sites, potentially resulting in a greater auxin influx (Mazarei et al., 2003). LAX genes together with AUX1 are important during nematode feeding sites initiation as single mutants show no effect on infection but the double *aux1/lax3* and quadruple mutant *aux1/lax1/lax2/lax3* display a marked decrease in infection. Additionally, the effector protein 19C07 of *H. schachtii* interacts with LAX3 and is thought to assist in increasing auxin influx and induce numerous cell wall–remodelling enzymes (Lee et al., 2011). During RKN infection the expression of AUX1 and LAX3 is increased and likely, also leads to an increase of auxin similar to CN infections (Kyndt et al., 2016).

The role of ABCB efflux carriers remain elusive in the infection process, but interestingly transcriptomic data from *A. thaliana* roots suggest that these differentially expressed transporters are generally downregulated (5/9) upon infection by *H. schachtii* and *M. incognita* (Supplementary Table 1). This suggests that these efflux carriers are downregulated to prevent the establishment of a uniform auxin distribution. This in turn would keep the auxin maxima localised at the feeding site and prevents it from dissipating.

Overall it seems that CN and RKN manipulate both influx and efflux carriers to induce an auxin accumulation in feeding sites. PIN proteins show complex alterations in expression and localisation patterns to facilitate auxin accumulation, whereas auxin importers are upregulated upon infection ABCB transporters are downregulated to prevent auxin leakage. The precise mechanisms by which efflux and influx carriers can be manipulated by nematodes remain unknown, but the two nematode effectors discussed and changes in hormonal pathways seem to be relevant.

## Auxin Perception in Nematode Feeding Sites Through the TIR1-AFB Signalling Cascade

Root-knot nematodes and cyst nematodes likely employ various means to generate and maintain an auxin maximum in their feeding site (Goverse et al., 2000; Grunewald et al., 2009). These elevated levels of auxin result in the transcription of a large set of genes that enact their function and contribute to the infection. In Arabidopsis, a family of 23 DNA-binding AUXIN RESPONSE FACTOR (ARFs) transcribe auxin-responsive genes (Li et al., 2016). In the absence of IAA, these transcription factors are inhibited through dimerisation with a member of the auxin/indole-3-acetic acid (Aux/IAA) family. In the presence of IAA the inhibitor is ubiquitinated by an E3 ubiquitin ligase SKIP, CULLIN, F-BOX (SCF) complex and subsequently degraded (Salehin et al., 2015). IAA facilitates the binding between the Aux/IAA and the F-box gene from the SCF complex that is part of the TRANSPORT INHIBITOR RESPONSE1/AUXIN SIGNALING F-BOXs (TIR1/AFBs) receptor family (Salehin et al., 2015). As such, the subsequent activation of the ARF transcription factors is called the TIR1-AFB signalling pathway.

Other auxins can activate this TIR1-AFB signalling pathway in varying degrees. The auxin IPA does not significantly interact with the TIR1-AFB pathway with only a very weak binding and with a noticeably more rapid dissociation rates (Uzunova et al., 2016). IBA does not activate the IAA signalling cascade as the molecule possesses a long side chain that makes it unable to adopt a conformation for binding to the TIR1–Aux/IAA co-receptor pocket (Uzunova et al., 2016). The auxin 4-Cl-IAA binds to the TIR1 auxin receptor (Jayasinghege et al., 2019) and is able to activate the signalling cascade with at least partial overlap in gene activation (Johnstone et al., 2005). As 4-Cl-IAA can activate the same signalling cascade as IAA it was expected that the response to the former would result in similar activation of gene transcription as that for IAA. However, 4-Cl-IAA stimulates gibberellin biosynthesis and regulates the ethylene response in a manner that IAA cannot (Jayasinghege et al., 2017). Similarly, PAA activates the TIR1-AFB signalling pathway and has many overlapping primary response genes with IAA (Sugawara et al., 2015). However, all major processes regulated by PAA can also be regulated by IAA and its functional difference from IAA is attributed to its difference in transport. Activation of the TIR1-AFB signalling complex by any auxin results in the transcription of primary auxin response genes.

The primary auxin response genes can be categorised into three major groups: Aux/IAA, GRETCHEN HAGEN 3 (GH3), and small auxin up RNA (SAUR) (Abel and Theologis, 1996). The Aux/IAA family functions as transcriptional repressors of auxin-response genes but are also one of the first major groups to be upregulated upon auxin signalling. This negative feedback loop results in a complex regulatory mechanism of IAA signalling given the flexibility in the binding specificity of Aux/IAA to a particular ARF (Luo et al., 2018). The GH3 family of acyl-acid-amido synthetases catalyse the conjugation of amino acids to various endogenous hormones among which is auxin (Chen et al., 2010). IAA-amino acid conjugates are essential for maintaining auxin homeostasis as they can function as either degradation intermediates or storage forms (Woodward and Bartel, 2005). The SAUR family is the largest group of early response genes and was originally identified in a differential hybridisation screen for genes that were rapidly induced by auxin in elongating soybean hypocotyl sections (McClure and Guilfoyle, 1987). SAUR genes contain no known biochemical motif to indicate their function but have a variety of different roles in cellular, physiological, and developmental processes (Ren and Gray, 2015). Of note here is the capability of some SAURs to regulate auxin transport, which is suspected to occur through either elevated plasma membrane H^+^-ATPase activity or Ca^2+^ regulation due to the CaM-binding activity of those SAURS (Ren and Gray, 2015).

For *H. schachtii* it has been shown that nearly all ARFs are active in syncytia and their expression patterns vary among infection stages (Hewezi et al., 2014). A select group of ARFs (3, 6, 10–12, 14, 15, 20–22) seems to be expressed in the syncytium during early feeding site initiation, while a different set of ARFs (1, 2, 4, 5, 7, 9, 17–19) is expressed in neighbouring cells. Transcriptomic databases reveal that ARF4, ARF6, ARF19, and ARF1A1C are also expressed during *M. incognita* infection in *A. thaliana* roots, but the precise timeframe and localisation remain unclear (Supplementary Table 1). LATERAL ORGAN BOUNDARIES-DOMAIN (LBD) genes are a family of transcription factors transcribed by various ARF proteins and play a crucial role in the growth and development of plants (Lee et al., 2015). *LBD16* is an example of a downstream signalling gene that is expressed in RKN feeding sites and is coincidentally induced through both IAA and PAA (Cabrera et al., 2014b; Sugawara et al., 2015; Olmo et al., 2017). *LBD16* is induced by either ARF7 or ARF19 and functions as a transcription factor leading to the divisions in the xylem pole pericycle for lateral root formation. Its importance in root-knot nematode feeding site formation is demonstrated by the *35S:LBD16-SRDX* overexpression line in which no feeding sites could be established and by the *lbd16* knockout mutant which shows a lower infection rate (Cabrera et al., 2014b; Olmo et al., 2017). The result of LBD16 repression by the SRDX protein is more severe than that of the *lbd16* knockout but this is potentially explained by the fact that the *35S:LBD16-SRDX* transgene might also repress the transcription of other LBD16 homologs, thereby exacerbating the effect. In contrast, *LBD16* and its co-regulated genes seem to be selectively repressed in feeding sites of the cyst nematode H. *schachtii* (Cabrera et al., 2015b). In addition, neither an overexpression mutant or a SRDX repression line seems to have any effect on the infection rate (Cabrera et al., 2014b).

Regulation of ARF expression is required in nematode feeding sites, given their distinct expression profiles. The major functional regulator of ARF activity is the Aux/IAA repressor family. As a primary auxin response group, several Aux/IAA genes are upregulated in nematode feeding sites (Supplementary Table 1). It has been shown that the effector protein 10A07 of *H. schachtii* physically interacts with Aux/IAA16 (IAA16) and presumably inhibits its function to promote auxin signalling (Hewezi et al., 2015). This hypothesis is supported by the fact that mutants of IAA16 (*IAA16*) and IAA7 (*axr2*), a protein from the same clade, in *A. thaliana* roots are highly susceptible to CNs (Goverse et al., 2000; Hewezi et al., 2015). Interestingly, a mutant of another closely related protein, *IAA14* (*SLR*) in *A. thaliana* results in a more resistant plant (Grunewald et al., 2008). Mutants of the Aux/IAA genes IAA12 (*BDL*) and IAA28 (*IAR2*) in *Arabidopsis thaliana* additionally show a RKN resistant phenotype (Olmo et al., 2020). Another regulator of ARF is AUXIN REPRESSED PROTEIN (ARP). It has been shown that ARP1 can act as a repressor of plant growth and an activator of disease resistance by regulating the expression of ARF8 (Zhao et al., 2014). The *ARP* gene in a wild peanut relative, *Arachis stenosperma* is upregulated during infection by *M. arenaria* (Guimaraes et al., 2010). In this case it likely functions as a regulator of ARF expression during infection.

As previously discussed activity of the primary auxin response group GH3 can be detected in feeding sites early on in the infection of white clover (*Trifolium repens* cv. Haifa) by *M. javanica* (Hutangura et al., 1999). PAA induces, similarly to IAA, the expression of several GH3 (*GH3.2*, *GH3.3*, *GH3.4*, and *GH3.5*) and Aux/IAA (*IAA1*, *IAA2*, *IAA5*, and *IAA13*) genes (Sugawara et al., 2015). Although PAA has been found in the in callus tissue infected with *Bursaphelenchus xylophilus* its presence in CN or RKN feeding sites has yet to be reported (Kawazu et al., 1996; Zhang et al., 1997).

The last primary auxin response group, SAUR, is likewise differentially expressed in nematode feeding sites and the majority of differentially expressed SAUR genes are downregulated during infection (24/38) (Supplementary Table 1). Little is known about the role of individual SAURs during nematode infection, however, given their potential to regulate auxin transport a better understanding of SAURs might give insight in how nematodes affect PIN localisation during infection. The relation between SAURs and PINs is still unclear, but it has been shown in *A. thaliana* that PIN2 is crucial for the proper functioning of SAUR19-mediated root waving (Spartz et al., 2012).

**FIGURE 4:**
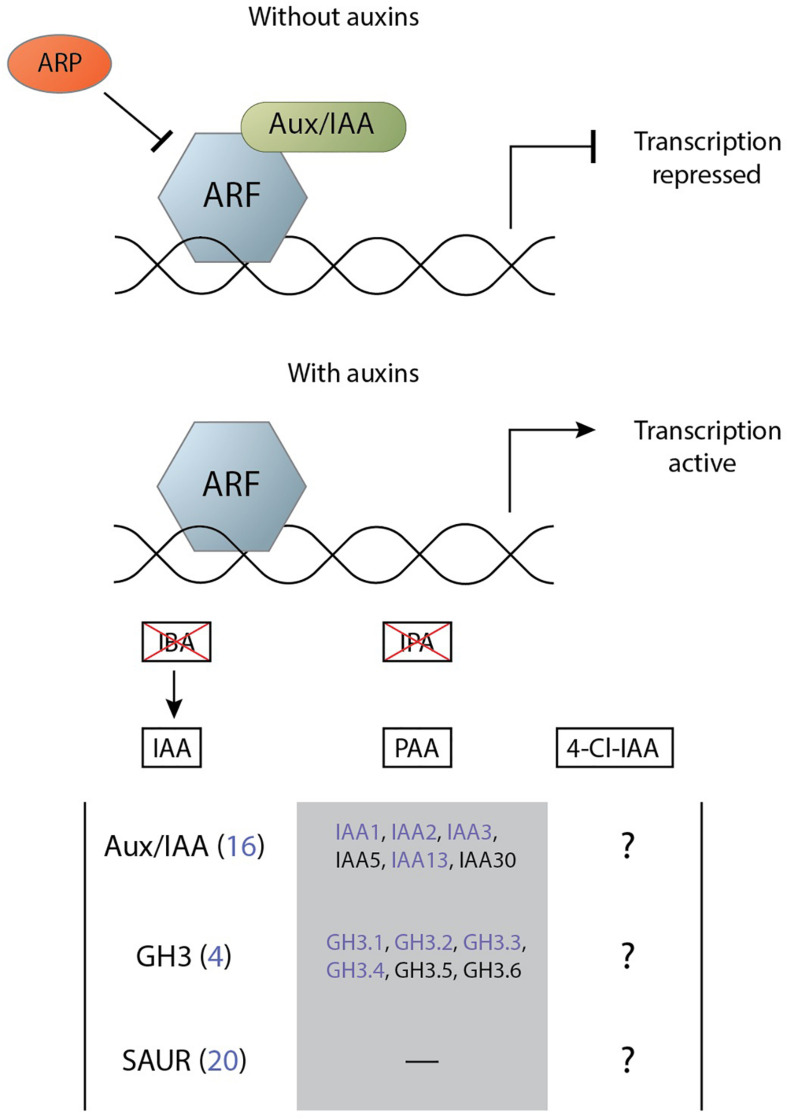
Simplified mechanism of auxin perception through the TIR1-AFB signalling cascade leading to the activation of primary auxin response genes. Transcription of auxin response genes is repressed by the Aux/IAA and ARP proteins (top). The presence of the auxins PAA, IAA, and 4-Cl-IAA degrade Aux/IAA and allow for gene transcription by ARF (bottom). The effect of the auxin IBA on transcription by ARF is mediated via conversion into IAA and the auxin IPA does not significantly activate the signalling pathway. Members of three primary response gene families can be activated by this system, of which various members are differentially regulated in feeding sites of cyst and root-knot nematodes (indicated in purple) (Supplementary Table 1). There is partial overlap between the genes that can be activated by PAA and those that are activated by IAA. On the other hand, it is unknown which primary response genes 4-Cl-IAA can activate.

Overall, genes of the major primary response groups GH3, SAUR, and Aux/IAA seem to be involved in RKN and CN infection (Figure 4). ARF genes become active and exhibit distinct spatial expression patterns in the infection site. ARF activation leads to the transcription of all three known primary response groups. The transcription of the Aux/IAA group gives in turn another layer of complexity to the regulation of auxin-mediated ARF signalling. In addition, GH3 genes become active likely to regulate hormone levels and the SAUR family, although active, remains an unknown factor in the infection process.

## Auxin Signalling in Nematode Feeding Sites Through Non-TIR1-AFB Pathways

Perception of IAA above a certain threshold activates the TIR1-AFB signalling cascade, which in turn triggers the expression of a set of auxin-responsive genes. However, not all auxins activate this signalling pathway and instead affect plant development through alternative methods. So, auxins may affect gene transcription during nematode infection through such alternative pathways.

Of the five naturally occurring auxins the ones that are indicated to act outside the classical signalling cascade are 4-Cl-IAA, IPA, and IBA. Although 4-Cl-IAA can activate the TIR1-AFB signalling pathway, it stimulates gibberellin biosynthesis and regulates ethylene responses independent from the TIR1-AFB signalling pathway (Jayasinghege et al., 2017). In pea, the ethylene response is regulated by 4-Cl-IAA through the expression of ethylene biosynthesis genes such as *1-aminocyclopropane-1-carboxylate oxidase* (*ACO) 1*, *ACO2* and *ACO3*. These genes catalyse the final step in the biosynthesis pathway and convert 1-aminocyclopropane-1-carboxylic acid (ACC) to ethylene. The notion that IAA induces ethylene in feeding sites has long been proposed and 4-Cl-IAA could play a role here as well. This holds true specifically for the plant species in the phylogenetic clades of the Fabeae and Trifoleae from the Fabaceae family where 4-Cl-IAA is found.

In contrast, the hormone IPA is unable to properly activate the TIR1-AFB signalling pathway and its molecular mechanisms in plants remain unknown (Simon et al., 2013). However, IPA inhibits primary root elongation and induces secondary root formation at high concentration in *A. thaliana*. In addition IPA failed to significantly activate the *DR5rev:GFP* reporter line supporting its inability to activate the TIR1-AFB signalling pathway. IPA functions as a potent antioxidant by reacting with hydroxyl radicals at a controlled diffusion rate (Poeggeler et al., 1999). Hydroxyl radicals are members of ROS and act as core regulators in a sophisticated network of signalling pathways in plants (Bhattacharjee, 2012). ROS plays an important role in plant defence against pathogens, such as nematodes, through the induction of a hypersensitive response, followed by programmed cell death (O’Brien et al., 2012). *M. javanica* prevents this response using the effector TTL5 which interacts with a ferredoxin-thioredoxin reductase catalytic subunit (AtFTRc) in Arabidopsis. This interaction increases the hosts ROS scavenging activity and suppresses host resistance (Lin et al., 2016). Similarly, the nematode might suppress programmed cell death through localised accumulation of IPA that acts there as a ROS scavenger.

Indole-3-butyric acid seems to be widespread in plants and has been detected in Arabidopsis, tobacco, pea, maize, and potato (Korasick et al., 2013). Additionally, it has been detected in the feeding sites of M. *incognita* infecting *A. thaliana* (Yu and Viglierchio, 1964). IBA is unable to activate the TIR1-AFB signalling cascade and although IBA can be converted to IAA a role for IBA in plant development outside of this conversion has also been speculated. IBA seems to be more effective in plant rooting and propagation than IAA. Furthermore, IBA is capable of restoring both lateral rooting and gravitropism in the *lrt1* rice mutant, while IAA only seems to rescue the former (Chhun et al., 2003). Therefore, it has been speculated that IBA can function as a signalling molecule independent of IAA (Ludwig-Müller, 2000) but no concrete signalling pathway has been identified.

Overall, the hormones 4-Cl-IAA, IPA and IBA might affect nematode infection through non-TIR1-AFB signalling pathways. For IPA this might be in the form of an antioxidant, while for 4-Cl-IAA this might be through activating ethylene biosynthesis. IBA strongly induces adventitious root formation and as secondary root formation is a process often observed during RKN and CN infection the presence of IBA in the feeding sites of *M. incognita* suggests a role for this hormone in the formation of secondary roots during infection.

## Conclusion and Perspectives

**FIGURE 5:**
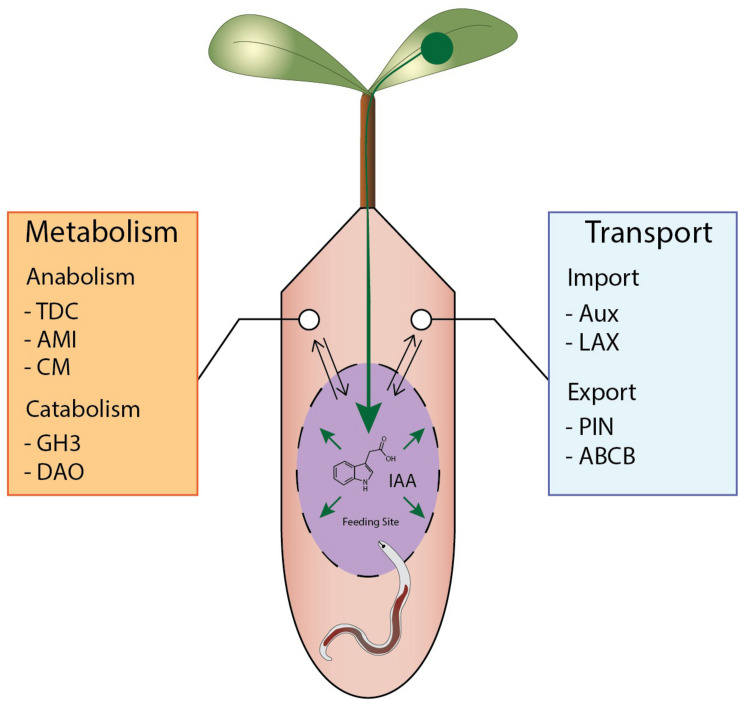
Overview of potential mechanisms leading to IAA accumulation during nematode feeding sites formation. A schematic feeding site is depicted in purple with auxin flows shown in green. IAA accumulates due to regulation of auxin transport redirecting IAA to the feeding site from the vascular bundle. In addition, local IAA synthesis occurs in and/or around the feeding site while auxin catabolism is repressed.

In this review we evaluated if and how different auxins could play a role during nematode infection and how auxin related processes are changed upon infection (Figure 5). Bioassays and chemical tests indicated already in the early 60s that IAA is of importance in feeding cell development induced by root-knot and cyst nematodes. However, other auxins, such as IBA (Kawazu et al., 1996; Zhang et al., 1997), may also accumulate during nematode infection. Clearly, examining the presence of IBA, PAA, 4-Cl-IAA, and IPA in a range of plant species with state-of-the-art metabolomics is worthwhile to clarify the role of auxins in feeding sites. Also studying the precursors of auxins with novel technologies is essential to uncover the pathways. In feeding sites, the IAOx pathway has the most support for *de novo* IAA synthesis to occur through (Figure 2). Surprisingly, no intermediate metabolites have been reported for the IPyA pathway which is the main auxin biosynthesis route in plants. The IAOx pathway is mainly found in members of the Brassicaceae family and as such manipulation of that pathway would only be relevant in that family. Therefore, it would be interesting to study if different auxin biosynthesis pathways are induced by different nematodes in non-Brassicaceae species.

Expression data and overexpression studies indicate that degradation and conjugation are involved in controlling and containing auxin levels in infected tissues (Supplementary Table 1). The old concept that peroxidases inactivate auxin and IAA oxidases are regulated by phenolic compounds seems not valid anymore due to new insights and therefore, it is more likely that enzymes such as DAO tune auxin levels. The early upregulation of conjugation enzymes seems contradictory as elevated auxin levels are required for proper feeding site initiation. However, the gene family responsible for auxin conjugation is also involved in the catabolism of other plant defence related hormones such as JA and SA (Chen et al., 2010). An alternative explanation is that the early upregulation of conjugation enzymes is required to dampen auxin signalling in neighbouring cells, while a high level of auxin is maintained in the actual feeding cell. It is noted that the interpretation of expression data is often hampered by the absence of data exclusively obtained from feeding cells without contamination from surrounding cells. A potential way to resolve this might be to use single cell sequencing technology or laser ablation to specifically study processes in nematode feeding sites (Strock et al., 2019).

Influx and efflux dynamics of IAA at the cellular level changes drastically upon nematode infection, but the underlying regulatory mechanisms remain actually unknown (Grunewald et al., 2009; Kyndt et al., 2016). Differences in intercellular transport mechanisms between auxins may add an additional regulatory layer to reprogram plant cells into feeding sites. While this review gives some examples of possible regulators of intercellular IAA transport via PINs it might be worthwhile to investigate the expression and changes in vesicle transport and PIN internalisation in feeding sites in more detail. The finding that sedentary nematodes secrete the plant hormone cytokinin might provide a clue in this process, since this plant hormone is shown to regulate both PIN expression and localisation during lateral root formation.

Local accumulation of auxin in the feeding sites of both CN and RKN leads to TIR1-AFB-mediated signalling. This perception of auxin has been shown mainly using transcriptomic data and via the activation of the DR5 promotor in localisation studies. However, this does not prove that solely IAA is present as both PAA and 4-Cl-IAA can activate the same signalling pathway. Data on the role of any other auxin during nematode infection beside IAA is grossly underrepresented, while it might be able to shed light on important infection events such as nematode feeding site initiation and development. Another relatively unexplored research area is the biological role and significance of the various components of the TIR1-AFB signalling cascades in nematode infection of which only few have been studied in detail.

Overall, sedentary nematodes induce various processes in their host that result in the accumulation of IAA and potentially other auxins at their feeding site during infection. These nematodes stimulate local IAA biosynthesis, regulate auxin catabolism, and modify IAA transport. Achieving this auxin accumulation is an import step in the life cycle of the nematode and having multiple methods to this goal provides a robust infection mechanism. CN and RKN both possess the means to modify the various routes leading to auxin accumulation, albeit in a different manner, as is clearly demonstrated by their difference in regulation of IAA transport through PIN. Identifying what role other auxins play in the infection process and identifying the tools each nematode uses to manipulate auxin homeostasis will bring us closer to understanding how nematodes develop their feeding sites.

## Author Contributions

JB and AG contributed to conception and design of the study. JL-T provided RNA-seq data. MO wrote the first draft of the manuscript. All authors contributed to manuscript revision, read, and approved the submitted version.

## Conflict of Interest

The authors declare that the research was conducted in the absence of any commercial or financial relationships that could be construed as a potential conflict of interest.
